# The recombinant spike S1 protein induces injury and inflammation in co-cultures of human alveolar epithelial cells and macrophages

**DOI:** 10.1371/journal.pone.0318881

**Published:** 2025-02-10

**Authors:** Yanru Liu, Hong Yu, Jia He, Jianyin Li, Denggao Peng

**Affiliations:** 1 Department of Emergency Medicine, Shenzhen Third People’s Hospital, Second Hospital Affiliated to Southern University of Science and Technology, Shenzhen, Guangdong Province, China; 2 Department of Pathology, Shenzhen Third People’s Hospital, Second Hospital Affiliated to Southern University of Science and Technology, Shenzhen, Guangdong Province, China; 3 Department of Internal Medicine, Shenzhen Third People’s Hospital, Second Hospital Affiliated to Southern University of Science and Technology, Shenzhen, Guangdong Province, China; Lerner Research Institute - Cleveland Clinic, UNITED STATES OF AMERICA

## Abstract

The current lack of a straightforward and convenient modeling approach to simulate the onset of acute lung injury (ALI) has impeded fundamental research and hindered the screening of therapeutic drugs in coronavirus disease 2019 (COVID-19). The co-cultured human pulmonary alveolar epithelial cells (HPAEpics) and alveolar macrophages (AMs) were exposed to the complete medium, three concentrations of recombinant spike S1 protein (0.1, 1, and 10 μg/mL), or lipopolysaccharide (LPS) (10 μg/mL). The cells were harvested at 1, 2, and 3 days post-exposure. Lactate dehydrogenase (LDH) release, and IL-6, TNF-ɑ, and malondialdehyde (MDA) production were quantified and compared. Compared to those exposed to medium, co-cultures of HPAEpics and AMs exposed to a concentration of S1 protein at 10 μg/mL demonstrated significantly increased levels of LDH release (22.9% vs. 9.1%, and 25.7%), IL-6 (129 vs. 74, and 110 pg/mg of protein), and TNF-ɑ (75 vs. 51, and 86 pg/mg of protein) production, and similar to those exposed to LPS. However, no statistically significant differences were observed in MDA production. Compared to those harvested at 1 or 2 days post-exposure, co-cultured cells harvested at 3 days post-exposure exhibited increased levels of LDH release (23.4% vs. 14.9%, or 16.7%), IL-6 (127 vs. 81, or 97 pg/mg of protein) and MDA (5.6 vs. 3.2, or 3.8 nmol/mg of protein) production, but exhibited lower TNF-ɑ (58 vs. 79 pg/mg of protein) production than those harvested at 2 days post-exposure. After 3 days of exposure, co-cultures of HPAEpics and AMs showed significantly increased levels of LDH release (25.3% vs. 18.4%), and MDA production (5.5 vs. 4.3 nmol/mg of protein) compared to HPAEpics monocultures, and increased levels of LDH release (25.3% vs. 13.8%), IL-6 (139 vs. 98 pg/mg of protein) and MDA (5.5 vs. 4.7 nmol/mg of protein) production, and decreased TNF-ɑ (59 vs. 95 pg/mg of protein) production compared to AMs monocultures. **Conclusions:** The exposure to a concentration of S1 protein at 10 μg/mL in co-cultures of HPAEpics and AMs induced significant injury and inflammation three days post-exposure. This methodology for establishing a COVID-19-associated ALI model may have promising potential applications and value.

## Introduction

The pathophysiology and pathogenesis of acute lung injury (ALI) or its more severe form, acute respiratory distress syndrome (ARDS), induced by severe acute respiratory syndrome coronavirus 2 (SARS-CoV-2), remain incompletely elucidated despite their status as a leading cause of mortality in coronavirus disease 2019 (COVID-19) patients [1, 2]. The most distinguishing characteristic of ALI/ARDS in COVID-19 is diffuse alveolar damage (DAD) triggered by a cytokine storm, which manifests predominantly as consolidation, ground-glass opacity, or haziness in chest radiographs [2, 3]. Currently, there are no effective treatment options available for this condition in clinical practice [3, 4]. Therefore, it is crucial to establish an appropriate disease model, explore its pathogenesis from multiple perspectives while identifying novel and efficacious therapeutic targets.

Animal models of host-pathogen interactions are indispensable for comprehending the pathogenesis of ALI/ARDS and evaluating treatment efficacy, necessitating their refinement to closely resemble disease features observed in humans [5, 6]. It is widely acknowledged that SARS-CoV-2 utilizes human angiotensin converting enzyme 2 (ACE2) as its receptor for cellular entry [7], while exhibiting low affinity towards murine ACE2 in rodents that are commonly used and easily accessible in experimental settings. The utilization of transgenic mice expressing human ACE2 may thus present a viable solution to address this issue [8, 9]. However, translating knowledge from rodent studies into human clinical applications can be challenging. In general, the practical application of animal models for studying COVID-19 has the following disadvantages: conducting experiments with live SARS-CoV-2 can pose difficulties and hazards, requiring the use of biosafety level 3 (BSL-3) laboratories [5]; executing experimental procedures requires advanced technical expertise and complex conditions; it is relatively costly and time-consuming.

In vitro cell models, based on human primary or immortalized cells, align with the ethical objective of reducing the utilization of transgenic mice and non-human primates. They enable the investigation of specific cellular targets that are not feasible in vivo [5]. Similar to tissue culture, organs-on-chips, and organoids, primary cell models entail higher costs, more intricate modeling methodologies, and elevated technical demands. These demands include expertise in cell culture techniques and meticulous control of experimental conditions, thereby restricting their ubiquitous adoption in conventional laboratories [5, 10]. The utilization of immortalized cell lines offers several advantages, including consistent availability, reduced laboratory maintenance costs, the capacity for multiple passages, and the mitigation of ethical concerns associated with the use of human tissue. However, utilizing a single cell line for modeling is inadequate to accurately represent the complex interplay of multiple physiological processes involved in DAD. Several studies have reported that human alveolar epithelial cells (AECs) and alveolar macrophages (AMs) may induce excessive inflammatory responses and oxidative stress while eliminating pathogens through host-pathogen interactions and cell-to-cell interactions after infection [11, 12]. In this study, we established a more straightforward and convenient co-culture model using two immortalized cell lines to adequately mimic the structural and physiological characteristics of human lung tissue. Furthermore, we hypothesized that substituting live SARS-CoV-2 with non-infectious recombinant spike S1 protein could potentially induce injury and inflammation in this co-culture system, which comprises human AECs and AMs.

## Materials and methods

### Cell cultures

Human pulmonary alveolar epithelial cell (HPAEpic, BFN608007335) and human AM (BFN607200566) cell lines were employed in our study to represent the AECs and AMs, modeling the morphological and pathological features of human COVID-19 pneumonia. Surface markers expressed by HPAEpics include aquaporin 5 (AQP5), which is mainly expressed in type I AECs, and surfactant protein C (SP-C), which is mainly expressed in type II AECs. AMs express cluster of differentiation (CD) 68, CD11c, and other markers associated with macrophage activity, such as human leukocyte antigen-DR (HLA-DR) and CD80/CD86. These cell lines were purchased from Bluefbio (Shanghai) Biotechnology Development Co., Ltd. Lipopolysaccharide (LPS, from Escherichia coli 055:B5, purified by phenol extraction) was sourced from Sigma-Aldrich. The SARS-CoV-2 spike S1 protein (Source: Recombination; Protein Tag: His-tag; Purity > 90%, as determined by Coomassie blue stained SDS-PAGE, free of LPS contamination) was acquired from Shanghai Bohu Biotechnology Co., Ltd. The frozen cell line was rapidly thawed by immersing it directly in a 37°C warm water bath and agitating until completely melted. Subsequently, the cell suspension was aspirated using a pipette, transferred to a centrifuge tube, and thoroughly mixed with complete medium composed of Dulbecco’s Modified Eagle Medium (DMEM, Gibco 11960, Life Technologies, Grand Island, NY, USA) supplemented with 10% fetal bovine serum (FBS, Hyclone in Logan, UT, USA). Following centrifugation at 1000 revolutions per minute for 10 minutes, the supernatant was discarded and the cell pellet at the bottom of the centrifuge tube was resuspended. The resulting cell suspension was then transferred to a T25 culture bottle after counting and adjusting the cell density using complete medium. Finally, incubation took place at 5% carbon dioxide and 37°C until cells reached approximately 80% confluence before passage. In subsequent passages or experiments, the complete medium of T25 culture bottle was aspirated, lightly flushed with PBS buffer for 3 times, and 0.25% EDTA pancreatic enzyme solution was added for digestion for 2 minutes. After that, the cells were washed off with the complete medium, centrifuged, and the supernatant was discarded to collect cells.

**Fig 1:**
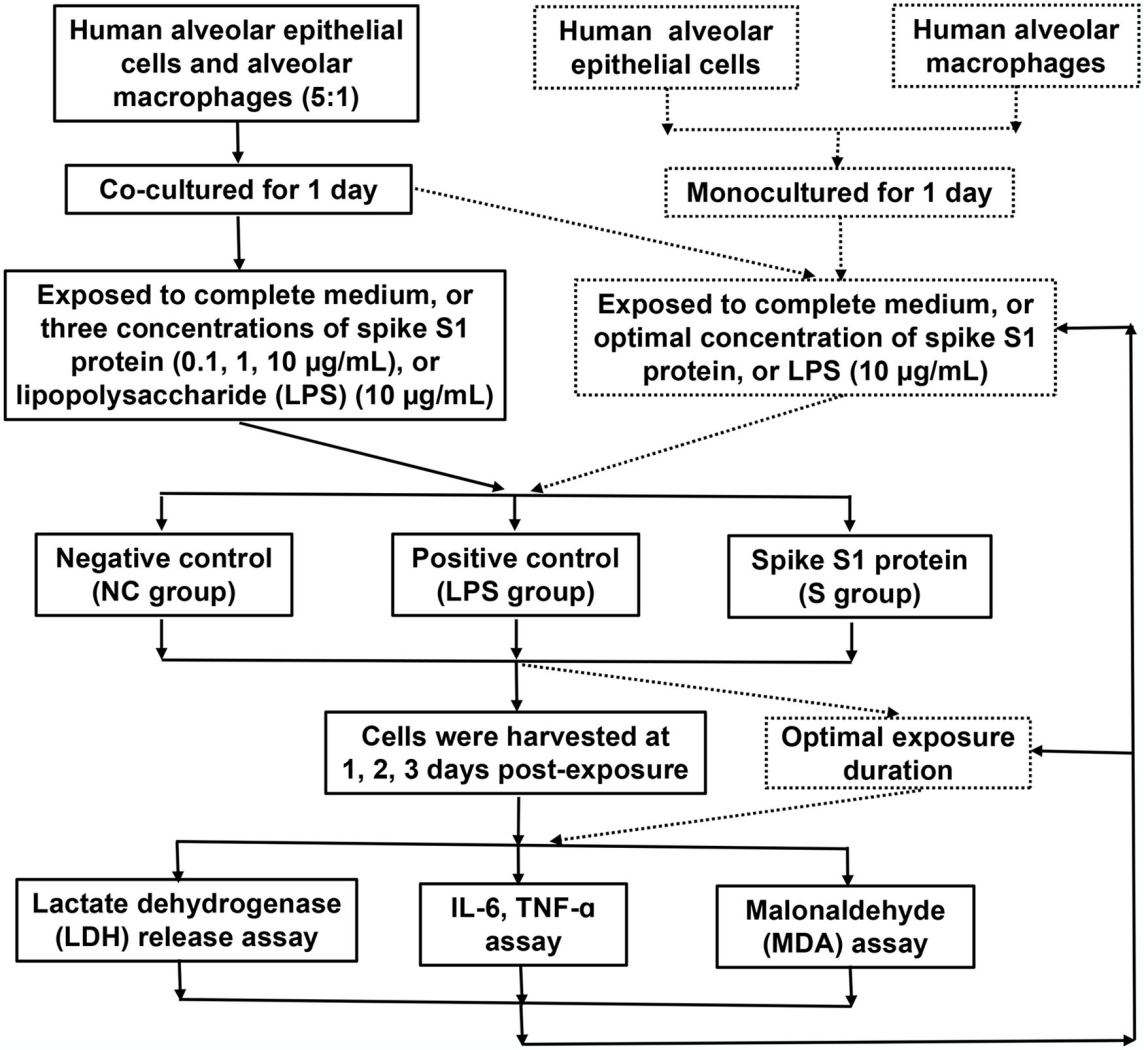
The study design and timeline for culture treatment.

Referring to previous literature reports [13], AMs and HPAEpics were co-cultured into 48-well plates at a ratio of 1:5. Unbiased treatment group assignment was guided by an online random number generator (www.random.org). After 24 hours, cell co-cultures were exposed to the complete medium (negative control, NC), or three concentrations (0.1, 1, and 10 μg/mL) of S1 protein (named S_0.1, S_1, and S_10, respectively), or 10 μg/mL of LPS (positive control). The co-cultured cells were harvested at 1, 2, and 3 days post-exposure. Notably, considering that the interpassage period is about 2 days, in order to avoid injury caused by excessive cell density, the co-cultured cells harvested at 1, 2, and 3 days post-exposure were initially composed of 0.5, 0.4, 0.3 × 10^5^ AMs and 2.5, 2, 1.5 ×10^5^ HPAEpics, respectively. The optimal concentration and duration of exposure to S1 protein are defined as the concentration and duration at which the most significant injury and inflammation could be induced in cell cultures based on the study objective. Either HPAEpics (× 10^5^) or AMs (× 10^5^) monocultures were exposed to the complete medium, or optimal concentration of S1 protein, or 10 μg/mL of LPS; and were collected at optimal harvest time. The study design and timeline for culture treatment were visually depicted in Fig 1.

### Outcome measures

Cultures were inspected and injuries were estimated under phase contrast microscopy before exposure and harvesting. All researchers conducting these assays were blinded to treatment groups. To reduce the risk of random results, the test was repeated three times for each sample, and the average of the three measurements was calculated to determine the final value. Given the scientific rigor and the reproducibility and confidence of the results, all experiments in this study were repeated at least three times.

### Bicinchoninic Acid (BCA) protein quantitative assay

The Enhanced BCA Protein Assay Kit was purchased from Shanghai Gene-Optimal Science & Technology Co., Ltd. The absorbance of cell lysates (0.1% Triton X-100) in each well at a wavelength of 595 nm was quantified using a Thermo Scientific Microplate Reader, and the total protein content was determined using the standard curve and the volume utilized.

### Thinprep liquid-based cytology test (TCT)

The ZEISS A1 optical microscope and the LG300D-700 self-imaging system were used to evaluate and analyze the pathology and morphology of cell cultures based on TCT. One well of each group of cell cultures per condition after exposure was randomly selected by an online random number generator (www.random.org). All cells from the selected well were extracted and quickly transferred into a 10 mL centrifuge tube containing Red Preservative (CytoRich™, USA) for full mixing, then left for static and fixed for 30 minutes at room temperature. After centrifugation (5 minutes, 2500–3000 rpm), the supernatant was discarded, and the cell deposits were aspirated and added to the settling chamber of the production rack for sedimentation for 10 minutes to make a thin layer of cell smear, which was stained with haematoxylin and eosin (H&E). Each cell smear was evaluated at a 400 × objective with 10 images of different locations, from which one image with the most significant cell injury was selected for qualitative description.

### Lactate dehydrogenase (LDH) release assay

The extent of cell injury was subsequently quantified using the LDH release assay, which offers an accurate measure of cell death in this model that correlates well with more laborious and bias-prone cell counts. The release of LDH from the cell’s cytoplasm to the culture medium occurs as a result of cellular membrane damage and subsequent cell lysis. The quantification was based on evaluating LDH’s capacity to catalyze the oxidation of lactic acid into pyruvic acid, which is contingent upon an increase in its extracellular release level. The augmentation in LDH activity observed in the supernatants of cell cultures indicates a positive correlation with the proportion of non-viable cells (increased cytotoxicity is associated with a higher abundance of deceased cells). According to the instructions provided by YEASEN Biotech Co., Ltd for the LDH Cytotoxicity Assay Kit, colorimetry was employed to measure the absorbance at a wavelength of 490 nm in the supernatant of each well culture, enabling quantification of LDH activity. The mean LDH activity in background wells (complete medium) from the same plating was subtracted from all values to determine the cytotoxicity-induced activity. Subsequently, the LDH activity in each well was divided by the mean maximum LDH activity determined in the cell lysates (Cell Lysis Buffer) of sister cultures to calculate the percentage of LDH release. In order to combine data prepared from different plates and experiments, which may vary somewhat in cell number and density, all values were normalized based on the ratio of total protein in each well to the average total protein per well from the same plating.

### Enzyme-linked immune-sorbent assay (ELISA) and colorimetry

The levels of soluble mediators in the supernatant of cell cultures were quantified using commercially available interleukin-6 (IL-6) and tumor necrosis factor-ɑ (TNF-ɑ) ELISA kits (Wuhan BOSTER Biological Engineering Co., Ltd). These kits employed sandwich ELISA technology, where the absorbance of the sample at a wavelength of 450 nm was measured by a Thermo Scientific Microplate Reader. The malondialdehyde (MDA) test Kit (Nanjing Jiancheng Bioengineering Research Institute Co., Ltd) was based on colorimetry. MDA in lipid peroxide degradation products in cell lysates can be condensated with thiobarbituric acid to form a red product with maximum absorption peak at 532 nm. To account for potential variations in cell number and density among wells and under different experimental conditions, the obtained results for IL-6, TNF-ɑ, and MDA were normalized to the total protein content in each well. Therefore, all final values by ELISA or colorimetry were expressed as pg/mg or nmol/mg of total proteins.

### Statistical analysis

The data were analyzed and figures were generated using GraphPad Prism 9.0 software. Continuous variables were summarized as mean with standard deviation (SD) and presented as mean ± SD. Two-way analysis of variance (ANOVA), fitting a full model (main column effect, main row effect, and column/row interaction effect), was used to assess differences between groups, and *P* values were adjusted using Bonferroni correction for multiple comparisons. A significance level of *P* < 0.05 was considered statistically significant in all applied tests.

## Results

### Effects of concentration and duration of exposure to S1 protein on co-cultures of HPAEpics and AMs

**Fig 2:**
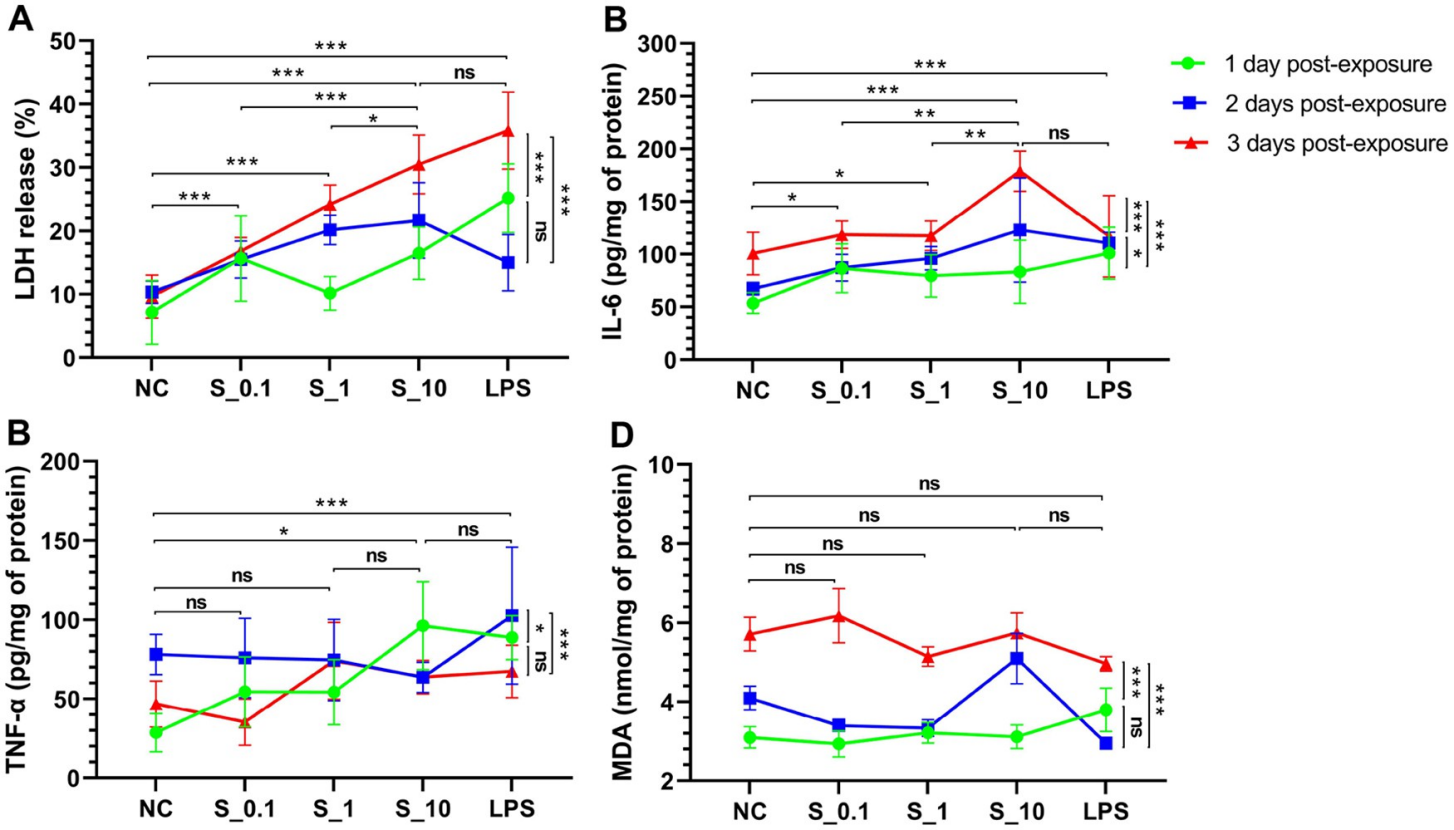
Effects of S1 protein and exposure duration on co-cultures of HPAEpics and AMs. AM, alveolar macrophage; HPAEpic, human pulmonary alveolar epithelial cell; IL-6, interleukin-6; LDH, lactate dehydrogenase; LPS, lipopolysaccharide; MDA, malonaldehyde; NC, negative control; TNF-ɑ, tumor necrosis factor-ɑ. Symbols and error bars on the connecting lines in the line chart represent means and standard deviations. Co-cultured cells were exposed to complete medium (NC), or three concentrations (0.1, 1, and 10 μg/mL) of S1 protein (S_0.1, S_1, and S_10), or 10 μg/mL of LPS (positive control); and were harvested at 1, 2, and 3 days post-exposure. Two-way analysis of variance was used, *P* values were adjusted with Bonferroni correction for multiple comparisons, n = 6 per condition. *P < 0.05, **P < 0.01, ***P < 0.001, ns, no significant. ^a^Main exposure effect; ^b^main exposure duration effect; ^ab^interaction. (A) LDH release, ^a^*P* < 0.001, ^b^*P* < 0.001, ^ab^*P* < 0.001. (B) IL-6 production, ^a^*P* < 0.001, ^b^*P* < 0.001, ^ab^*P* < 0.01. (C) TNF-ɑ production, ^a^*P* < 0.001, ^b^*P* < 0.001, ^ab^*P* < 0.001. (D) MDA production, ^a^*P* = 0.10, ^b^*P* < 0.001, ^ab^*P* < 0.05.

The co-cultured HPAEpics and AMs (n = 6 per condition) exposed to S1 protein at a concentration of 10 μg/mL demonstrated significantly increased levels of LDH release (averages of the three exposure duration groups: 22.9% vs. 9.1%, or 16%, or 18.2%, and 25.7%) (Fig 2A), IL-6 production (averages of the three exposure duration groups: 129 vs. 74, or 98, or 98, and 110 pg/mg of protein) (Fig 2B) compared to those exposed to the complete medium, 0.1 μg/mL or 1 μg/mL of S1 protein, and similar to those exposed to 10 μg/mL of LPS; and TNF-ɑ production (averages of the three exposure duration groups: 75 vs. 51, and 86 pg/mg of protein) (Fig 2C) compared to those exposed to the complete medium, and similar to those exposed to LPS. However, no statistically significant differences were observed in MDA production. Compared to those harvested at 1 or 2 days post-exposure, co-cultured cells harvested at 3 days post-exposure exhibited increased levels of LDH release (averages of the five exposure groups: 23.4% vs. 14.9%, or 16.7%) (Fig 2A), IL-6 (averages of the five exposure groups: 127 vs. 81, or 97 pg/mg of protein) (Fig 2B) and MDA production (averages of the five exposure groups: 5.6 vs. 3.2, or 3.8 nmol/mg of protein) (Fig 2D), but exhibited lower TNF-ɑ production (averages of the five exposure groups: 58 vs. 79 pg/mg of protein) (Fig 2C) than those harvested at 2 days post-exposure. Taken together, the optimal concentration for exposure to the S1 protein was found to be 10 μg/mL, and the optimal exposure duration in this model was 3 days post-exposure.

### Pathology and morphology of cell injury after exposure to S1 protein or LPS

**Fig 3:**
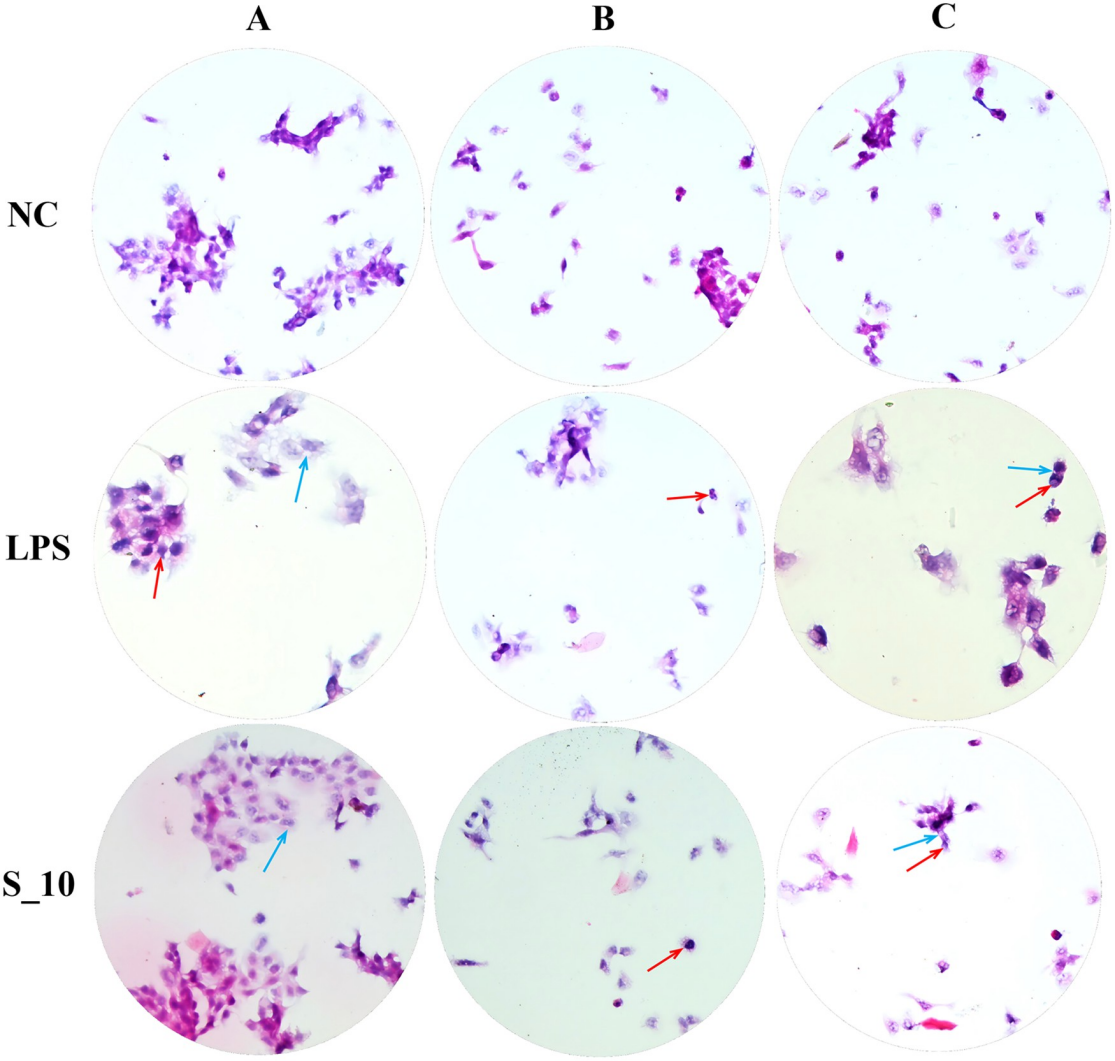
Pathology and morphology of cell injury in co-cultures or monocultures 3 days after exposure to S1 protein or LPS. AM, alveolar macrophage; HPAEpic, human pulmonary alveolar epithelial cell; LPS, lipopolysaccharide; NC, negative control. Three exposure groups, NC, S_10, LPS, were exposed to complete medium, or S1 protein (10 μg/mL), or LPS (10 μg/mL). Images of cultured cells under optical microscope (400 x) were based on thinprep liquid-based cytology test, stained with H&E. The pathology and morphology changes of cell injury included that the cytoplasm was swollen, dissolved, and reduced (blue arrow), and the nucleus became smaller, degenerated, vacuolated, and irregular, with a wrinkled nuclear membrane (red arrow). (A) Monocultured AMs. (B) Monocultured HPAEpics. (C) Co-cultured HPAEpics and AMs.

After exposure to 10 μg/mL of S1 protein or 10 μg/mL of LPS, the pathology and morphology changes of cell injury in monocultured AMs (Fig 3A), monocultured HPAEpics (Fig 3B), co-cultured AMs and HPAEpics (Fig 3C) included that the cytoplasm of the cell was swollen, dissolved, and reduced, and the nude nucleus was produced; the nucleus became smaller, degenerated, vacuolated, and irregular, with a wrinkled nuclear membrane; a few cells have broken nuclei without cytoplasm.

### Effects of cell co-culture or monoculture on injury and inflammation

**Fig 4:**
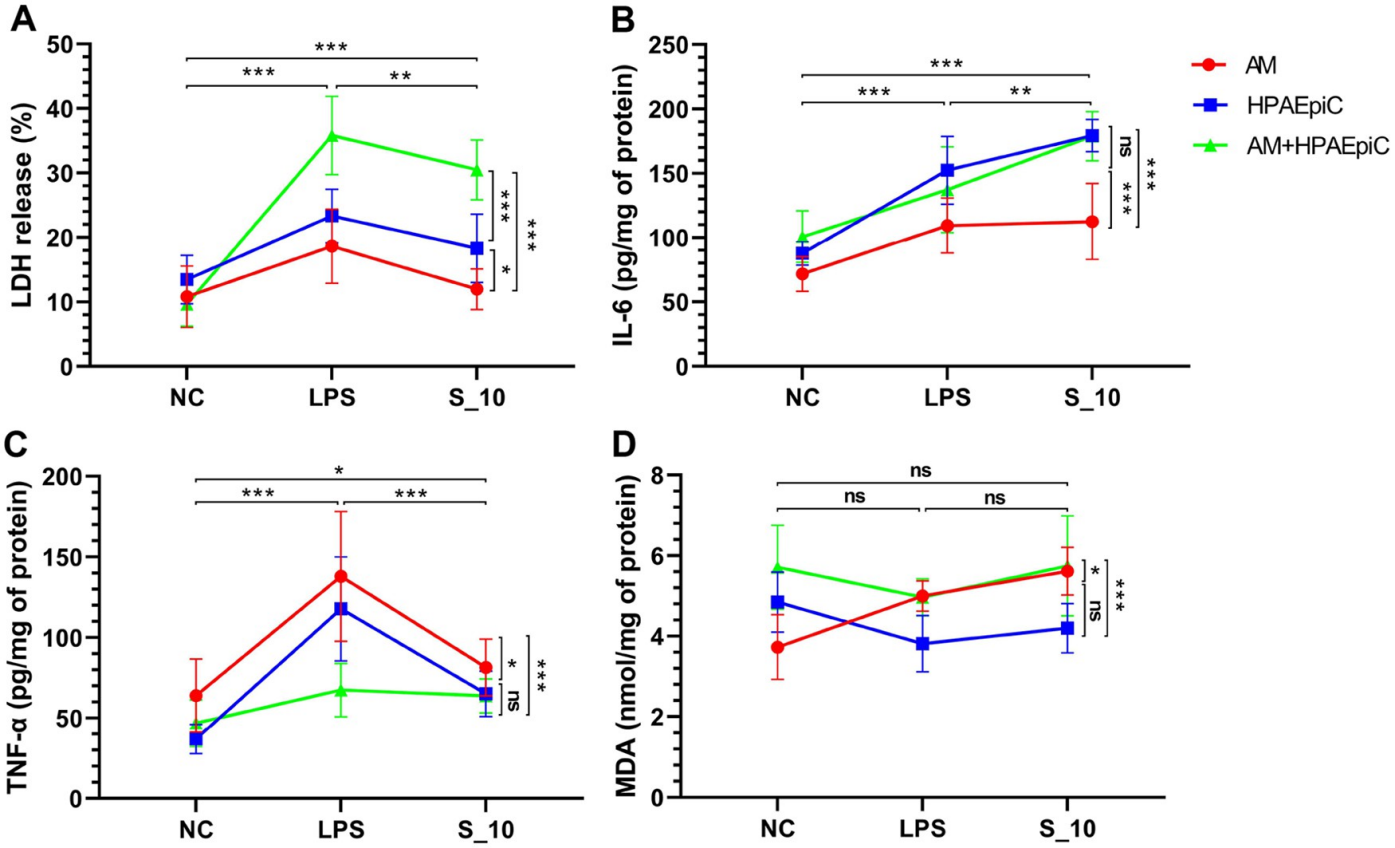
Effects of cell culture patterns on injury and inflammation induced by the S1 protein. AM, alveolar macrophage; HPAEpic, human pulmonary alveolar epithelial cell; IL-6, interleukin-6; LDH, lactate dehydrogenase; LPS, lipopolysaccharide; MDA, malonaldehyde; NC, negative control; TNF-ɑ, tumor necrosis factor-ɑ. Symbols and error bars on the connecting lines in the line chart represent means and standard deviations. Three exposure groups, NC, LPS, and S_10, were exposed to complete medium, LPS (10 μg/mL), or S1 protein (10 μg/mL). Cells were harvested at 3 days post-exposure. Two-way analysis of variance was used, *P* values were adjusted with Bonferroni correction for multiple comparisons, n = 6 per condition. *P < 0.05, **P < 0.01, ***P < 0.001, ns, no significant. ^a^Main exposure effect; ^b^main culture pattern effect; ^ab^interaction. (A) LDH release, ^a^*P* < 0.001, ^b^*P* < 0.001, ^ab^*P* < 0.001. (B) IL-6 production, ^a^*P* < 0.001, ^b^*P* < 0.001, ^ab^*P* < 0.05. (C) TNF-ɑ production, ^a^*P* < 0.001, ^b^*P* < 0.001, ^ab^*P* < 0.01. (D) MDA production, ^a^*P* = 0.13, ^b^*P* < 0.001, ^ab^*P* < 0.01.

Co-cultures and monocultures of HPAEpics and/or AMs (n = 6 per condition) after 3 days of exposure to 10 μg/mL of S1 protein showed significantly increased levels of LDH release (averages of the three culture pattern groups: 20.3% vs. 11.3%, and 25.9%) (Fig 4A), IL-6 production (averages of the three culture pattern groups: 157 vs. 57, and 133 pg/mg of protein) (Fig 4B), and TNF-ɑ production (averages of the three culture pattern groups: 70 vs. 49, and 108 pg/mg of protein) (Fig 4C) compared to those exposed to the complete medium, and similar to those exposed to LPS. However, no statistically significant differences were observed in MDA production (Fig 4D). After 3 days of exposure, co-cultures of HPAEpics and AMs showed significantly increased levels of LDH release (averages of the three exposure groups: 25.3% vs. 18.4%) (Fig 4A), and MDA production (averages of the three exposure groups: 5.5 vs. 4.3 nmol/mg of protein) (Fig 4D) compared to HPAEpics monocultures, and increased levels of LDH release (averages of the three exposure groups: 25.3% vs. 13.8%) (Fig 4A), IL-6 (averages of the three exposure groups: 139 vs. 98 pg/mg of protein) (Fig 4B) and MDA production (averages of the three exposure groups: 5.5 vs. 4.7 nmol/mg of protein) (Fig 4D), and decreased TNF-ɑ production (averages of the three exposure groups: 59 vs. 95 pg/mg of protein) (Fig 4C) compared to AMs monocultures.

## Discussion

The study provides novel information and innovative approaches that may be relevant for modeling COVID-19-associated ALI. The modeling involved the following key steps: Firstly, a co-culture system was established by co-culturing HPAEpics and AMs in a 5:1 quantity ratio for 24 hours. Subsequently, the co-culture system was exposed to the recombinant SARS-CoV-2 spike S1 protein at a concentration of 10 μg/mL, followed by the induction of ALI three days post-exposure. The co-culture system can adequately simulate the structural and pathophysiological characteristics of the human lung. More importantly, the modeling method substitutes infectious live SARS-CoV-2 with a noninfectious recombinant spike S1 protein, which eliminates the requirement for a BSL-3 laboratory and effectively induces significant ALI while ensuring the safety of laboratory personnel by avoiding potential infections. Additionally, this approach offers significant advantages such as shorter experimental periods, improved cost-effectiveness, and the ability to expose cultures to disease-relevant stimuli under controlled conditions while also allowing for manipulation using a variety of techniques. It is well-suited for fundamental research on COVID-19-associated ALI/ARDS under conventional conditions and can be applied in therapeutic drug screening.

The spike protein, which is the fundamental component of the novel coronavirus, contains a receptor binding domain (RBD) located in its N-terminal residues. The RBD is responsible for binding to the ACE2 receptor on the surface of the host cell, facilitating subsequent viral invasion [9, 14]. The two subunits of the spike protein, namely S1 and S2, perform distinct functions: the S1 subunit primarily facilitates viral binding to host cell receptors, while the S2 subunit mediates fusion between the viral envelope and the host cell membrane. Consequently, the entry of SARS-CoV-2 necessitates the cleavage of the spike protein at the S1/S2 cleavage site, a process facilitated by furin, a protease enzyme present in host cells [15]. Notably, the recombinant SARS-CoV-2 S1 protein subunit vaccine has demonstrated immunogenicity by effectively stimulating a robust immune response and inducing high levels of neutralizing antibodies [16, 17]. Furthermore, specific antibodies targeting the S1-RBD have exhibited varying degrees of neutralization efficacy against multiple SARS-CoV-2 variants. These findings further emphasize the critical role played by the S1 subunit of the spike protein in both the infectivity and virulence of SARS-CoV-2, as well as in pathogen-host interactions.

Several studies have confirmed the potential of the SARS-CoV-2 spike protein to act as pathogen-associated molecular patterns (PAMPs), thereby triggering inflammatory responses through pattern recognition receptors (PRRs) expressed by innate immune cells, including macrophages [18–21]. These findings suggest that the spike protein or its S1 subunit can activate human monocytes/macrophages independently of virus replication, leading to the release of pro-inflammatory mediators and contributing to hyperinflammation in COVID-19 patients [18–20]. In contrast, the S2 subunit fails to elicit the same effect [21]. Our study observed a similar hyperinflammatory response and cellular injury induced by the recombinant spike S1 protein in co-cultured cells. Notably, the treatment effect of the S1 protein was observed to be significantly affected by its concentration. Compared with the complete medium, 0.1 μg/mL, 1 μg/mL, and 10 μg/mL of S1 protein induced higher LDH release, similar to 10 μg/mL of LPS, showing a roughly linear dose-response relationship. Interestingly, the exposure to a concentration of S1 protein at 10 μg/mL resulted in the most significant induction of LDH release and IL-6 production among the three S1 protein exposure groups. Furthermore, there was a significant interaction between the concentration and the duration of exposure to the S1 protein. Our model showed that the most significant cell damage and IL-6 production was achieved 3 days after exposure to 10 μg/mL of S1 protein, while TNF-α production peaked 1 day after exposure, which is significantly different from other studies where similar effects in human lung macrophages or peripheral blood mononuclear cells were observed within 24 hours and after exposure to lower concentrations of spike [20] or S1 protein [19]. However, this is consistent with the clinical natural course of cytokine storms (represented by IL-6) triggered by SARS-CoV-2 infection, and COVID-19-associated ALI/ARDS. Surprisingly, very complex oxidative stress was shown among groups with different treatments and/or different durations of exposure. In co-cultured cells, MDA production induced by LPS or three concentrations of S1 protein exhibited no difference compared with the complete medium, and was highest at 3 day post-exposure and lowest at 1 days post-exposure. The implications of these findings suggest that the exposure concentration and exposure duration should be carefully considered when treating in vitro cell cultures with spike proteins in the absence of a complex in vivo environment and persistent viral replication.

Macrophages are renowned for their critical role in initiating both innate and adaptive immunity to pathogens through the immune recognition of PAMPs by PRRs, as well as serving as a primary source of cytokines such as TNF-α and IL-6 that trigger immune and inflammatory responses. However, excessive production of these mediators contributes to the pathogenesis of ALI/ARDS [22, 23]. The air-blood barrier of the lung is primarily composed of AECs and AMs. AECs function as a diffusional barrier responsible for facilitating gas exchange and producing surfactant, while AMs serve as an immunological barrier, particularly against inhaled invading pathogens [11]. The coordination of action and interactions between AMs and AECs are crucial for maintaining the homeostasis of lung structure and function. In addition, neutrophils play an integral role in the lung’s antimicrobial defense by releasing pro-inflammatory cytokines and chemokines to recruit and activate other immune cells to infection or tissue injury sites, and by interacting with AMs [24]. The levels of LDH release, and MDA production post-exposure in co-cultures of HPAEpics and AMs were significantly elevated compared to those in monocultures of either HPAEpics or AMs. However, TNF-ɑ production was higher, and IL-6 production was lower in monocultures of AMs than co-cultures of HPAEpics and AMs, or monocultures of HPAEpics. These findings suggest that the cell-cell interactions between HPAEpics and AMs may play a key role in cell injury and inflammatory response, and that the production of inflammatory cytokines may be cell-specific. In addition, the pathology and morphology changes of cell injury based on TCT were observed in all cell cultures exposed to the S1 protein. Notably, the significant interaction between exposure to the S1 protein and cell culture patterns highlights the advantages of our model in simulating the onset of COVID-19-associated ALI.

Pyroptosis, apoptosis, necroptosis, and ferroptosis are major regulatory cell death pathways, contribute to the clearance of infected or potentially damaged cells, highlighting their importance in homeostasis and host defense against pathogens [25]. Ferroptosis is a distinctive form of cell death dependent on iron-dependent lipid peroxidation [26]. Multiple studies have shown that hyperferritinemia (defined as serum ferritin levels exceeding 400 ng/mL) in COVID-19 patients may not only indicate an acute phase reaction but also play a significant role in promoting the inflammatory cascade [27–30]. Circulating ferritin loses some of its iron content, producing high levels of highly reactive and potentially toxic free iron, which contributes to the formation of reactive oxygen species (ROS) induced by Fenton and Haber-Weiss reactions [27]. Imbalances in ROS production and clearance can cause oxidative stress damage to cellular biomolecules (lipids, nucleic acids, and proteins), which may initiate subsequent ferroptosis, exacerbating and promoting ALI/ARDS. Ferritin levels are significantly correlated with COVID-19 disease severity and adverse outcomes [28, 29]. Our previous meta-analysis also found that severe COVID-19 cases had higher levels of serum hepcidin and ferritin [31]. These findings suggest that iron dysmetabolism and ferroptosis may be involved in the pathogenesis of SARS-CoV-2-induced ALI/ARDS. In our model, although S1 protein similar to LPS can induce significant cell injury and the production of IL-6 and TNF-α, there is no significant difference in the production of MDA, so whether ferroptosis is the main mechanism of cell injury needs further investigation.

There are several limitations that warrant mention in our study. (1) The effects of exposure to different concentrations of S1 protein and different exposure duration on cell injury and inflammatory response in the monoculture of either HPAEpics or AMs were not investigated. (2) The co-culture ratio of AMs and HPAEpics at 1:5 is based on relevant literature reports, which may not be the most appropriate ratio in our experiment. (3) Co-cultured AMs and HPAEpics are passaged cell lines that may inevitably lose the functional diversity of primary cells. (4) The specific molecular mechanism of the S1 protein inducing cell injury and inflammatory response has not been investigated, and further experimental exploration is required. (5) The disadvantage of our model is the absence of neutrophils, which are known to play a significant role in the onset and development of SARS-CoV-2-induced ALI/ARDS. Consequently, these results may need to be further validated in animal models and should also be carefully interpreted and applied due to potential biases.

## Conclusions

The exposure to a concentration of S1 protein at 10 μg/mL in co-cultures of HPAEpics and AMs induced significant injury and inflammation three days post-exposure. This methodology for establishing a COVID-19-associated ALI model may have promising potential applications and value.

## Supporting information

S1 File Raw data and statistical analysis used in Figs 2 and 4. (XLSX)

## Abbreviations

ACE2: angiotensin converting enzyme 2
AEC: alveolar epithelial cell
ALI: acute lung injury
AM: alveolar macrophage
ANOVA: analysis of variance
AQP5: aquaporin 5
ARDS: acute respiratory distress syndrome
BCA: Bicinchoninic Acid
BSL-3: biosafety level 3
CD: cluster of differentiation
COVID-19: coronavirus disease 2019
DAD: diffuse alveolar damage
DMEM: Dulbecco’s Modified Eagle Medium
ELISA: enzyme-linked immune-sorbent assay
HLA-DR: human leukocyte antigen-DR
HPAEpic: human pulmonary alveolar epithelial cell
IL-6: interleukin-6
LDH: lactate dehydrogenase
LPS: lipopolysaccharide
MDA: malondialdehyde
PAMP: pathogen-associated molecular pattern
PRR: pattern recognition receptor
RBD: receptor binding domain
ROS: reactive oxygen species
SARS-CoV-2: severe acute respiratory syndrome coronavirus 2
SD: standard deviation
SP-C: surfactant protein C
TCT: thinprep liquid-based cytology test
TNF-ɑ: tumor necrosis factor-ɑ
